# miR-133b靶向*PKM2*基因对肺癌A549干细胞增殖及药物敏感性的影响

**DOI:** 10.3779/j.issn.1009-3419.2017.06.02

**Published:** 2017-06-20

**Authors:** 永华 米, 苗 何, 北忠 刘

**Affiliations:** 1 402160 重庆，重庆医科大学附属医院永川医院检验科 Department of Laboratory, Yongchuan Affiliated Hospital Chongqing Medical University, Chongqing 402160, China; 2 610500 成都，成都市新都区人民医院呼吸内科 Respiratory Medicine, Xindu District People's Hospital of Chengdu, Chengdu 610500, China

**Keywords:** MiR-133b, A549, PKM2, DDP, MiR-133b, A549, PKM2, DDP

## Abstract

**背景与目的:**

已有研究表明miR-133b可抑制肺癌细胞生长，miR-133b表达水平在肺癌组织及患者血清中显著降低，miR-133b通过靶向丙酮酸激酶M2型（pyruvate kinase isozyme type M2, *PKM2*）基因提高鳞癌细胞的放疗敏感性，但其机制尚未明了。本研究通过提取非小细胞肺癌细胞株A549的CD133^+^/CD34^+^干细胞，研究miR-133b对其增殖及顺铂类药物（cisplatin, DDP）敏感性的影响，探讨miR-133b与*PKM2*基因的关系，以及它们在肺癌干细胞中的作用。

**方法:**

使用miRBase和miRNAMap数据库进行miR-133b与*PKM2*基因的序列比对。应用免疫磁珠分选法从A549细胞中分选出CD133^+^/CD34^+^肺癌干细胞，流式细胞仪检测纯度。转染miR-133b进入CD133^+^/CD34^+^细胞，qRT-PCR检测验证miR-133b表达情况，CCK8法检测细胞增殖情况；15 μg/mL DDP处理转染miR-133b细胞，检测0 h、12 h、24 h、72 h的细胞凋亡情况；采用Western blot检测PKM2蛋白水平的表达。

**结果:**

*PKM2*基因可能为miR-133b的靶基因；流式结果显示CD133^+^/CD34^+^细胞的纯度为（92.15+4.27）%。qRT-PCR结果显示，与对照组相比，过表达miR-133b后，miR-133b明显上调，miR-133b抑制表达后，miR-133b明显下调（*P* < 0.05）。细胞增殖实验及Western blot结果显示，与对照组相比，miR-133b mimics组细胞的增殖能力显著降低（*P* < 0.05），PKM2蛋白水平明显降低（*P* < 0.05）；而抑制miR-133b表达则明显增加细胞的增殖能力及PKM2蛋白水平（*P* < 0.05）。DDP处理12 h后miR-133b mimics组细胞的凋亡持续明显高于对照组（*P* < 0.05）。miR-133b mimics+DDP组PKM2蛋白的表达明显低于对照组及miR-133b inhibitor组（*P* < 0.05）。

**结论:**

过表达miR-133b能够通过下调PKM2而抑制肺癌干细胞的生长和增殖，并且可以增强肺癌干细胞对DDP的敏感性。

## Abstract (en)

**Background and objective:**

It has been proven that miR-133b could inhibit cancer cell growth, the expression level of miR-133b was significant reduction in lung cancer tissue and serum of patients, and increase the radiation sensitivity of squamous cell carcinoma by targeting PKM2, but the exist mechanisms is not clear. The aim of this study is to explore the effect of miR-133b on proliferation in A549 lung cancer stem cells and drug sensitivity in DDP, and to explore the relationship between miR-133b and *PKM2* gene, as well as the effect of cancer stem cells.

**Methods:**

Using miRBase and miRNAMap database to sequence comparison miR-133b and *PKM2* gene. Using immune magnetic separation method to select the CD133^+^/CD34^+^ lung cancer stem cells from A549 cells, and using flow cytometry to detect the purity. The expression of miR-133b mRNA was detected by real-time fluorescence quantitative PCR (qRT-PCR). Cell proliferation was detected by CCK8 assay. 15 μg/mL DDP was treated to cells which was transfected with miR-133b, and apoptosis was detected by flow Cytometry at 0 h, 12 h, 24 h, 72 h. The expression of PKM2 protein was detected by Western blot.

**Results:**

Gene binding site report that *PKM2* gene may be the target gene of miR-133b; the results of flow cytometry showed that the purity of CD133^+^/CD34^+^ stem cells was (92.15±4.27)%. qRT-PCR results showed that compared with the control group, after overexpression of miR-133b, miR-133b was up-regulated and miR-133b was down regulated after miR-133b inhibition (*P* < 0.05). Compared with the control group, cell proliferation of miR-133b mimics group was significantly decreased (*P* < 0.05), PKM2 protein levels were significantly lower (*P* < 0.05); and cell proliferation of the miR-133b inhibitor group and PKM2 level was increased (*P* < 0.05). The apoptosis of miR-133b mimics group was significantly higher than that of control group (*P* < 0.05) after DDP treatment with 12 h. The expression of PKM2 protein in miR-133b mimics+DDP group was significantly lower than that in control group (*P* < 0.05).

**Conclusion:**

Overexpression of miR-133b can inhibit the growth and proliferation of lung cancer stem cells by down regulating PKM2, and can enhance the sensitivity of lung cancer stem cells to DDP.

微小RNA（microRNA, miRNA）是一类内源性、长度大约为18个-25个碱基的高度保守的非编码小分子RNA。可与靶mRNA的3′非翻译区（3′-UTR）特异性结合，在转录后水平调节基因的表达^[1]^，导致目标mRNA的降解或者基因沉默从而调节细胞分化、增殖、调亡、肿瘤发生等多种生物学过程。已有的研究^[2, 3]^表明miRNA能行使癌基因或抑癌基因的功能，miRNA参与和肿瘤的发生发展相关的许多重要生物学过程，包括肿瘤的凋亡、增殖、分化、转移、血管生成和免疫反应等。研究表明，miR-133b编码基因位于染色体臂6p^[4]^，在肺癌组织及患者血清中miR-133b呈低表达，通过刺激下游信号通路（Ras/Raf/MEK/ERK/MAPK、PI3K / AKT、STAT3、FAK等），促进了肿瘤细胞的增殖、血管生成、肿瘤侵袭转移及细胞凋亡的抑制^[5]^。刘等^[6]^研究表明，miR-133b与非小细胞肺癌（non-small cell lung cancer, NSCLC）辐射敏感度之间有一定的联系，miR-133b可通过靶向*PKM2*基因治疗抗放射性的肺癌。另有研究^[7]^报道miR-133a和miR-133b可以下调PKM2表达从而抑制舌鳞状细胞癌的增殖。本实验通过研究miR-133b与*PKM2*基因的关系及其对肺癌干细胞的作用，阐明miR-133b抗癌作用的部分机制，进一步证明miR-133b可作为一个潜在的抗癌新靶点。

## 1 材料与方法

### 1.1 材料

A549细胞株购自ATCC，DMEM购置于Invitrogen，miR-133b模拟物miR-133b mimics、阻遏物miR-133b inhibitor、miR-133b control购置于中国上海吉玛制药技术有限公司，CD133 MicroBead Kit（human）和CD34 MicroBead Kit（human）和购置于美国Miltenyi Biotec公司，转染试剂Lipofectamine 2000购置于Invitrogen公司，miRNA提取试剂盒mirVana RNA Isolation Kit，逆转录试剂盒Taqman miRNA Reverse Transcripation Kit购置于美国ABI公司，荧光实时定量PCR试剂盒、Annexin V、FITC凋亡检测试剂盒购置于BD公司，SYBR Green Master购置于瑞士罗氏公司，U6内参引物于上海生工公司设计，GAPDH一抗购置于Abcam公司，CCK-8试剂盒购置于南京凯基生物有限公司，流式细胞仪购置于BD公司。

### 1.2 细胞培养

细胞于DMEM培养基中培养，培养基中同时添加10%的胎牛血清，100 U/mL的青霉素、100 mg/mL的链霉素，置于37 ℃、含5%CO_2_的培养箱中培养。贴壁增殖至80%-90%融合时，用胰蛋白酶进行消化、传代。

### 1.3 基因结合位点报告

miR-133b与PKM2使用miRBase和miRNAMap数据库进行序列对比（网站：http://microrna.sanger.ac.uk，http://mirnamap.mbc.nctu.edu.tw）。

### 1.4 CD133^+^/CD34^+^肺癌干细胞的分选

收集培养至对数生长期的A549细胞，离心后加入Buffer重悬，加入磁珠标记的抗体（CD133和CD34），混匀后室温反应30 min，通过Auto MACS仪器，分选获得CD133^+^/CD34^+^细胞。采用流式细胞仪检测CD133^+^/ CD34^+^细胞的纯度。

### 1.5 转染及验证

#### 1.5.1 Lipofectamine 2000脂质体转染

miR-133b取对数生长期的A549干细胞，5×10^5^个/孔细胞接种于6孔板中，加入2 mL完全培养基，待细胞结合度约70%时进行转染。按照试剂盒说明书的操作方法，分别加入终浓度为100 nmol/L的miR 133b mimics、miR 133b inhibitor以及对照质粒miRNA con，每组设3个复孔进行转染，37 ℃放置48 h，更换为正常培养基培养，进行下一步实验。

#### 1.5.2 qRT-PCR检测miR-133b的表达

按照TRIzol试剂盒说明书提取细胞总RNA并逆转录为cDNA。miRNA cDNA采用miRNA 3'末端加Poly(A)法逆转录合成，具体步骤参照Taqman miRNA Reverse Transcripation Kit说明书。采用SYBR Green qPCR检测miR-133b（U6 snRNA为内参）的表达，利用2^-△△Ct^方法计算miR-133b的相对表达水平。PCR引物序列如下，U6 snRNA上游序列：5'-CTCGCTTCGGCAGCACA-3'，下游序列：5'-AACGCTTCACGAATTTGCGT-3'；miR-133b上游序列：5'-AAACCTGGCGGCCACGCTAC-3'，下游序列：5'-GACCGTGGTCCACTGCAGGC-3'。反应条件：95 ℃预变性2 s，95 ℃变性10 s，60 ℃退火20 s，70 ℃延伸1 s-10 s，扩增40个循环。

### 1.6 CCK8法检测细胞的增殖

#### 1.6.1 细胞增殖实验

分别检测转染6 d内A549干细胞的增殖情况。细胞以5×10^3^个/孔的密度接种在96孔板内，培养4 h后加入10 µL CCK-8，细胞培养箱内放置2 h后，使用酶标仪检测450 nm处检测各孔OD值，每组重复4次。相对增殖活力=实验组OD值/空白对照组OD值。

#### 1.6.2 细胞耐药性实验

转染48 h后，用15 μg/mL化疗药物DDP处理各组转染的CD133^+^/CD34^+^细胞0 h、24 h、48 h、72 h，收集各组各个时间点的细胞，流式细胞术检测细胞凋亡率。

### 1.7 Western blot检测蛋白表达

#### 1.7.1 转染对PKM2蛋白表达的影响

检测过表达及抑制miR-133b对A549干细胞蛋白水平的影响。取转染后的A549干细胞，裂解提取总蛋白。BCA法测定细胞裂解物的蛋白含量，取等量蛋白质以12%SDS-PAGE分离并转移至PVDF膜上，单克隆抗体4 ℃孵育过夜。PBST洗去一抗，HRP连接的二抗室温孵育2 h。洗涤，ECL试剂盒显色，凝胶成像系统采集成像。GAPDH作为内参。成像结果结合Image J灰度分析软件对PKM2蛋白水平进行分析对比。

#### 1.7.2 DDP处理对细胞PKM2蛋白表达的影响

检测过表达及抑制miR-133b的A549干细胞对在DDP药物处理后，PKM2蛋白水平的变化，取经15 μg/mL化疗药物DDP处理24 h的CD133^+^/CD34^+^细胞，提取总蛋白，Western blot结合Image J灰度分析软件分析其中PKM2蛋白水平，方法同上。

### 1.8 统计学分析

应用统计分析软件SPSS 17.0进行数据分析，实验结果采用平均数±标准差（Mean±SD）的方式表示，双向t检验来确定差异的显着性。*P* > 0.05表示差异无统计学意义；*P* < 0.05表示差异有统计学意义；*P* < 0.01表示差异具有显著统计学意义。

## 2 结果

### 2.1 基因结合位点报告以及流式结果

miRBase结果表明miR-133b有1, 038个靶基因结合位点，其中包括PKM2（数据库基因ID:ENST00000389093）。miR-133b和PKM2的基因结合位点1119-1136如图 1所示。流式结果如图 2所示，免疫磁珠分离纯化后CD133^+^/CD34^+^细胞纯度为（92.15+4.27）%。

**1:**
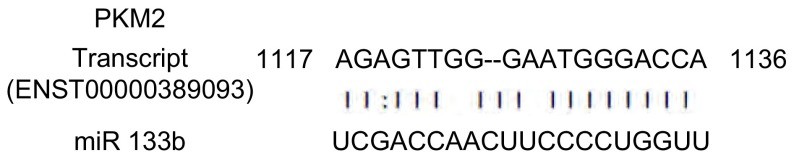
*PKM2*基因与miR133b基因结合位点报告 Binding sites of mature miR-133b on PKM2 transcript

**2:**
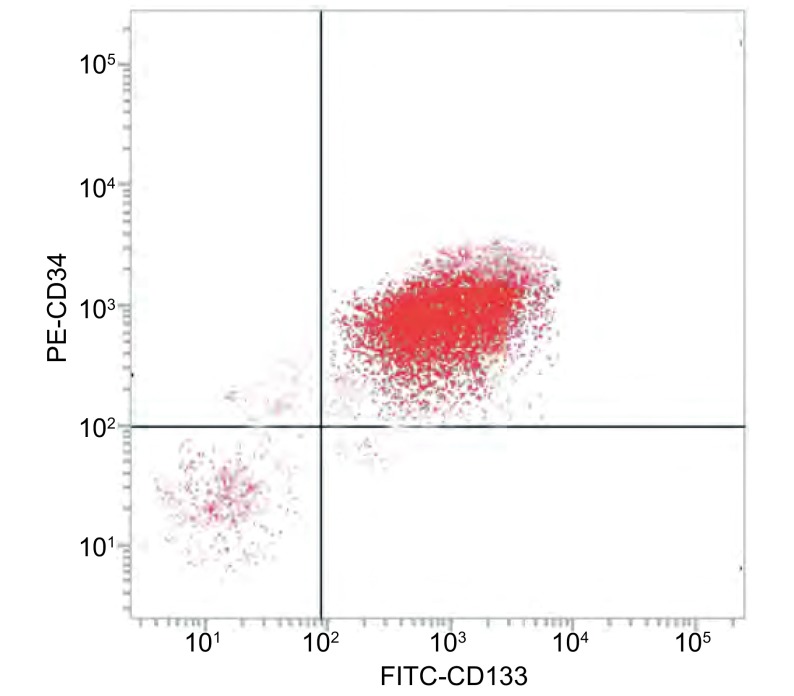
CD133^+^/CD34^+^细胞纯度检测 The purity of CD133^+^ /CD34^+^stem cells

### 2.2 qRT-PCR结果

qRT-PCR结果显示，与对照组相比，转染miR-133b mimics后，细胞内miR-133b表达明显上调（*P* < 0.01）；转染miR-133b inhibtor，细胞内miR-133b表达明显下调（*P* < 0.01），见图 3。结果提示转染成功，可有效干预miR-133b的表达。

**3:**
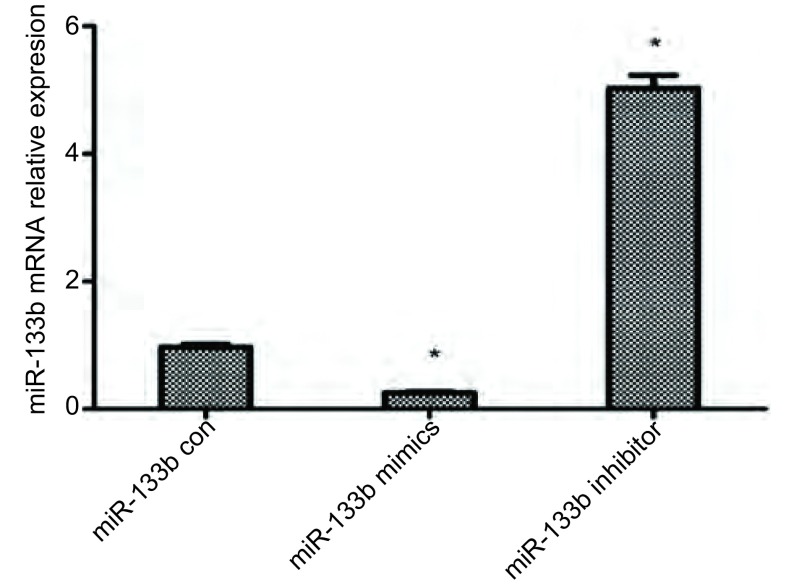
RT-PCR检测转染组miR-133b mRNA表达情况。^**^P < 0.001 *vs* 对照组 The expression of miR-133b in each group determined b real-time PCR. ^**^P < 0.001 *vs* control group

### 2.3 转染miR-133b对细胞增殖及PKM2蛋白表达的影响

CCK8增殖实验结果如图 4示，与对照组相比，过表达miR-133b后，CD133^+^肺癌干细胞的增殖能力显著降低（*P* < 0.05），在第4-6天下降最为显著；而抑制miR-133b表达细胞增殖能力明显增强（*P* < 0.05），在第3-4天增殖最为明显。Western blot结果如图 5所示，过表达miR-133b后CD133^+^肺癌干细胞PKM2蛋白的表达水平明显降低（*P* < 0.01）；而抑制miR-133b表达则明显增加CD133^+^肺癌干细胞PKM2蛋白水平（*P* < 0.01）。

**4:**
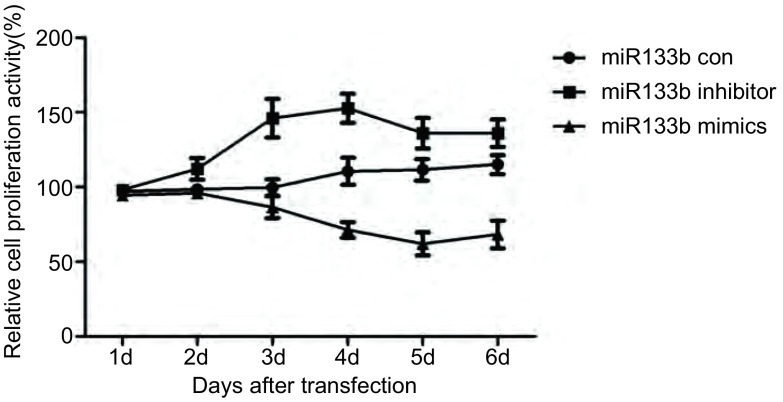
细胞增殖曲线 The curve of cell proliferation

**5:**
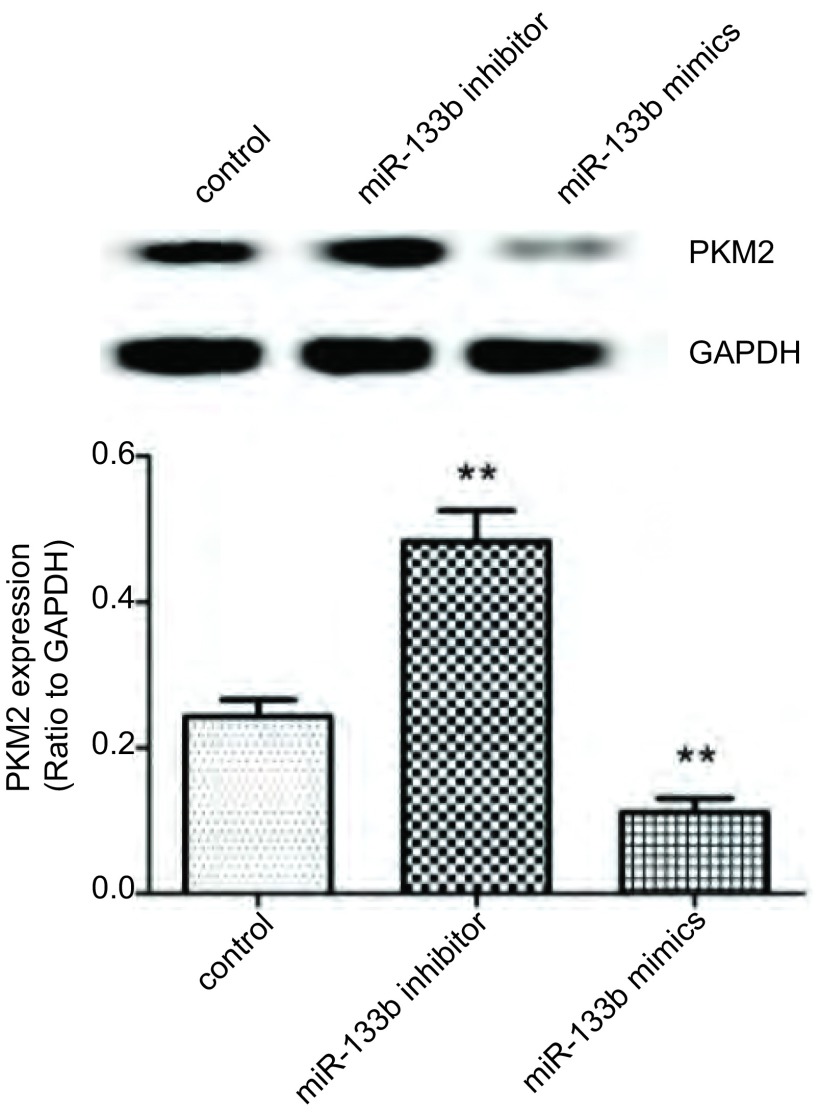
PKM2蛋白表达水平。^**^P < 0.001 *vs* 对照组 The expression levels of PKM2. ^**^P < 0.001 *vs* control group.

### 2.4 转染miR-133b对细胞凋亡的影响

细胞凋亡结果如图 6示，miR-133b mimics组细胞在DDP处理12 h后，细胞凋亡率明显高于对照组和miR-133b inhibitor组（*P* < 0.05），且miR-133b inhibitor组细胞凋亡率明显低于对照组，在DDP处理72 h差异更明显。

**6:**
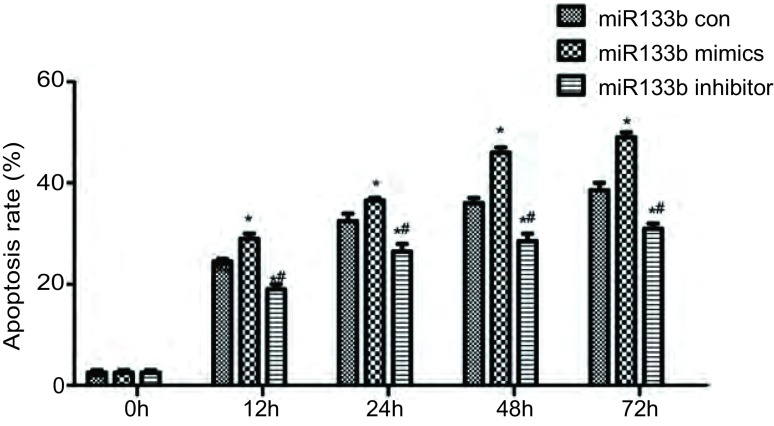
细胞凋亡率 Cell apoptosis rate

### 2.5 转染miR-133b与药物处理对PKM2蛋白表达的影响

Western blot结果如图 7所示，miR-133b mimics+DDP组PKM2蛋白的表达明显低于对照组及抑制miR-133b组（*P* < 0.05）。

**7:**
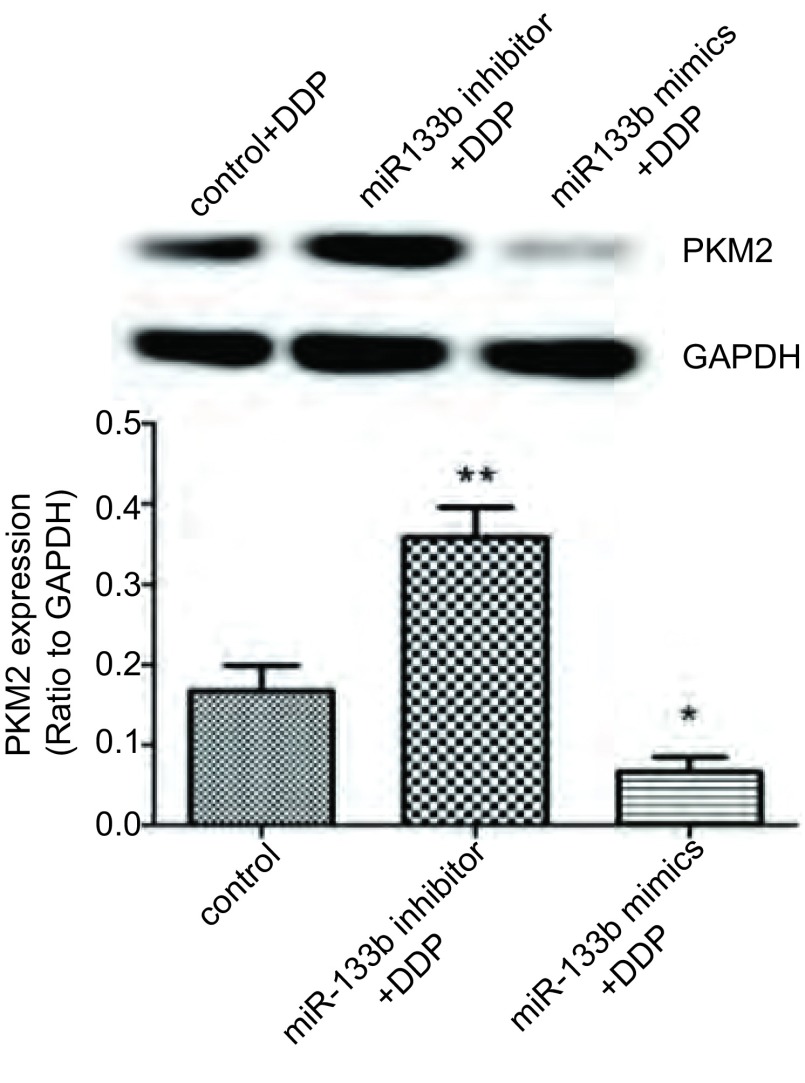
PKM2蛋白表达水平。^**^P < 0.001 *vs* 对照组，^*^P < 0.05 *vs* 对照组 The expression levels of PKM2. ^**^P < 0.001 *vs* control, ^*^P < 0.05 *vs* control.

## 3 讨论

肺癌的发病率与死亡率不断升高，已成为近年来的恶性肿瘤之首，严重威胁着人类的健康与生命^[8]^。尽管我们关于癌症的知识越来越多，但针对肺癌的有效治疗手段仍很有限。很多学者^[9, 10]^致力于鉴定肺癌中差异表达的miRNA并寻找其靶基因，以期为寻找新的治疗靶点提供重要的线索。

肿瘤干细胞是实体肿瘤中的一小部分，有增殖、自我更新、分化为肿瘤块的能力^[11]^。已有实验报道CD133为肿瘤干细胞特异标志分子，CD133^+^肿瘤干细胞由于具有干细胞的特性，其对化疗或放疗都具有不敏感性^[12]^。有研究^[13]^表明CD133、CD34和CD44可以作为分离人肺腺癌肿瘤干细胞的表面蛋白表标记组合。本研究利用CD133、CD34作为A549干细胞的特异性分子标记物，采用免疫磁珠分离出高纯度CD133^+^/CD34^+^肺癌干细胞。

研究^[14]^发现PKM2在多种恶性肿瘤中表达升高，且与肿瘤耐药性及预后相关，在肿瘤的发生发展中发挥重要的调控作用。PKM2以高活性四聚体和低活性的二聚体两种形式充当糖酵解双功能作用的传感器和调控器，确定其是代谢成乳酸还是合成大分子。PKM2四聚体形式具有丙酮酸激酶活性，能催化磷酸烯醇式丙酮酸转化为丙酮酸，与底物PEP有高亲和力，酶催化能力很强，为肿瘤细胞分化、增殖提供所需的能量。二聚体形式，则能催化合成细胞构建模块如核算、氨基酸、磷脂等^[15]^。*PKM2*是癌症治疗的一个重要的靶基因，PKM2在肝癌中高表达与患者的不良预后相关^[16]^。使PKM2高表达会促进癌细胞中间代谢产物的积累，对肿瘤治疗有益。有研究^[6]^表明，miR-133b通过靶向PKM2改善肺癌细胞的放疗敏感性。而本研究通过实验阐明miR-133b作为靶向PKM2的miRNA，两者之间的关系，及其抑癌作用。

我们使用miRBase和miRNAMap数据库进行分析，预测miR-133b与*PKM2*基因有靶向关系。有研究^[17, 18]^表明，CD133阳性细胞具有高度增殖分化潜能和体内成瘤能力及干细胞样特性。在肺癌组织及患者血清中miR-133b呈低表达，通过刺激下游信号通路（Ras/Raf/MEK/ERK/MAPK、PI3K/AKT、STAT3、FAK等），促进了肿瘤细胞的增殖、血管生成、肿瘤侵袭转移及细胞凋亡的抑制^[5]^。我们应用免疫磁珠分选法从A549细胞株中分选出CD133^+^/CD34^+^细胞，即A549干细胞，并应用流式细胞术检测其纯度。在CD133^+^/CD34^+^细胞的基础上转染miR-133b模拟物和阻遏物，运用qRT-PCR验证转染结果。之后利用CCK8法及Western blot检测miR-133b对CD133^+^/CD34^+^细胞增殖的影响，证明miR-133b可以靶向调节*PKM2*基因，影响肺癌干细胞增殖。为了阐明miR-133b对肺癌干细胞药物敏感性的影响，我们检测不同时间点的DDP对过表达及抑制miR-133b的CD133^+^/CD34^+^细胞的增殖情况的影响，发现miR-133b mimics+DDP组的细胞抵抗DDP的能力降低，细胞凋亡率明显升高。综上所述，我们发现过表达miR-133b可以有效降低CD133^+^/CD34^+^细胞的增殖能力，同时可以增强细胞对DDP的敏感性，并且这些作用均可能是通过靶向PKM2基因，影响PKM2的表达而体现的。为探索miR-113b对肺癌干细胞的作用机制以及网络调控奠定了基础，同时为肺癌的生物学防治提供了一个新的潜在靶标。
